# Bridging Single Neuron Dynamics to Global Brain States

**DOI:** 10.3389/fnsys.2019.00075

**Published:** 2019-12-06

**Authors:** Jennifer S. Goldman, Núria Tort-Colet, Matteo di Volo, Eduarda Susin, Jules Bouté, Melissa Dali, Mallory Carlu, Trang-Anh Nghiem, Tomasz Górski, Alain Destexhe

**Affiliations:** ^1^Department of Integrative and Computational Neuroscience (ICN), Centre National de la Recherche Scientifique (CNRS), Paris-Saclay Institute of Neuroscience (NeuroPSI), Gif-sur-Yvette, France; ^2^Department of Physics, Ecole Normale Supérieure, Paris, France

**Keywords:** computational neuroscience, neural network models, mean-field models, membrane biophysics, low-dimensional manifold, cerebral cortex, coupling, desynchronized

## Abstract

Biological neural networks produce information backgrounds of multi-scale spontaneous activity that become more complex in brain states displaying higher capacities for cognition, for instance, attentive awake versus asleep or anesthetized states. Here, we review brain state-dependent mechanisms spanning ion channel currents (microscale) to the dynamics of brain-wide, distributed, transient functional assemblies (macroscale). Not unlike how microscopic interactions between molecules underlie structures formed in macroscopic states of matter, using statistical physics, the dynamics of microscopic neural phenomena can be linked to macroscopic brain dynamics through mesoscopic scales. Beyond spontaneous dynamics, it is observed that stimuli evoke collapses of complexity, most remarkable over high dimensional, asynchronous, irregular background dynamics during consciousness. In contrast, complexity may not be further collapsed beyond synchrony and regularity characteristic of unconscious spontaneous activity. We propose that increased dimensionality of spontaneous dynamics during conscious states supports responsiveness, enhancing neural networks' emergent capacity to robustly encode information over multiple scales.

## Introduction

Brain activity transitions between healthy states, including stages of sleep, restful and aroused waking, as well as pathological states such as epilepsy, coma, and unresponsive wakefulness syndrome. From such a diversity of brain states, phenomenological categories encompassing similar spatio-temporal activity patterns can roughly, but usefully, be defined: unconscious (e.g., sleep and anesthesia) and conscious (e.g., waking and dreaming) brain states. At the macroscopic, global scale, unconscious brain states are dominated by high voltage, low frequency oscillatory brain activity related to the microscopic alternation of synchronous neuronal spiking and near silence (Steriade et al., 1993; Brown et al., 2010). Conversely, conscious states are macroscopically characterized by low voltage, high frequency, complex “disorganized” dynamics resulting from more asynchronous irregular (AI) microscopic network activity (Tsodyks and Sejnowski, 1995; Van Vreeswijk and Sompolinsky, 1996; Brunel, 2000), thought to be important for neural coding (Skarda and Freeman, 1987; Van Vreeswijk and Sompolinsky, 1996; Tononi and Edelman, 1998; Zerlaut and Destexhe, 2017).

Much as different states of matter like solids, liquids, and gases emerge from interactions between populations of molecules, different brain states may emerge from the interactions between populations of neurons. Statistical physics provides a mathematical framework to uncover structures of microscopic interactions underlying macroscopic properties. In this sense, macroscopically observed high synchrony, low complexity brain signals recorded from unconscious states may be accounted for by an increased coupling in the system's components, behaving more like a solid (Peyrache et al., 2012; Le Van Quyen et al., 2016; Olcese et al., 2016; Nghiem et al., 2018a). In contrast, conscious brain states may be described as higher complexity (Sitt et al., 2014; Engemann et al., 2018; Nghiem et al., 2018a), perhaps liquid-like.

Though quantitative expressions directly linking order and complexity are not straightforward, various definitions and metrics of complexity have been described to vary between brain states. Reports of enhanced complexity in conscious compared to unconscious states may be understood as increased dimensionality (El Boustani and Destexhe, 2010), namely the number of degrees of freedom needed to capture a system's dynamics. Intuitively, dimensionality relates, though is not reducible to, algorithmic complexity which quantifies the length of a deterministic algorithm required to reproduce an exact signal. For a random signal resulting from purely stochastic dynamics (similar to neural activity observed during conscious states), the length of the algorithm would be as long as the signal itself. In contrast, a purely oscillatory signal (reminiscent of unconscious brain dynamics) can be recapitulated by a shorter algorithm, easily described by a periodic trajectory in few dimensions.

**Figure 1:**
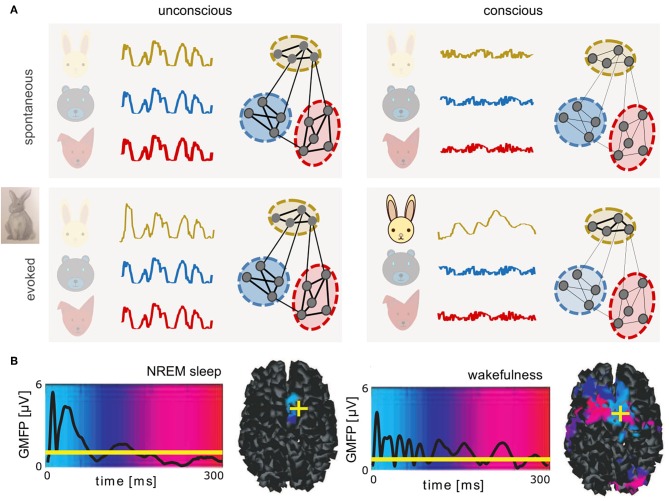
Complex dynamics associated with conscious brain states provide a potential substrate for neural coding. **(A)** Schematics of spontaneous (top) and evoked (bottom) dynamics in connected neuronal assemblies encoding different related concepts (different colors) in unconscious (left) and conscious (right) brain states. In unconscious brain states, slow, synchronous, large amplitude oscillations are observed. Stimuli delivered during unconscious states evoke large amplitude, transient responses similar to spontaneous activity. In contrast, during conscious states, asynchronous, irregular firing of neurons results in macroscopically desynchronized, low amplitude signals. Only networks recruited by the perturbation (here, a rabbit) produce lower-dimensional patterns that propagate relatively further in time and space. **(B)** Global mean-field power (GMFP) recorded with EEG in response to transcranial magnetic stimulation, during deep, non-rapid eye movement (NREM) sleep versus wakefulness. Mean EEG signal is represented by black traces. Background colors represent temporal latency (light blue, 0 ms; red, 300 ms) of maximum current sources, also shown in cortical space on the right, where yellow crosses represent the location of stimulation (right dorsolateral premotor cortex). Reprinted with permission from AAAS (Massimini et al., 2005). If brain dynamics between states may be described in analogy to states of matter, perturbing unconscious brains results in large, brief signals perhaps akin to a small perturbation of a solid, which can displace the solid briefly, but will not modify its internal structure. In contrast, the same perturbation delivered during conscious, liquid-like brain states results in smaller but more complex patterns that propagate further in time and space. Under this interpretation, in coding networks, responses evoked during conscious states could represent a form of transient “crystallization,” consistent with neural trajectories lying on low-dimensional manifolds.

Here, we aim to connect spatial scales from microscopic (nanometers to micrometers—molecules to whole neurons) to macroscopic brain activity (centimeters to meters—brain areas to individual subjects' brains), describing both spontaneous and evoked dynamics. Toward linking interpretations of studies between scales, mesoscopic data (micrometers to millimeters—populations of thousands to tens of thousands of neurons) have been useful to inform models of neuronal assemblies. The perspective concludes by discussing a hypothesis best tested with a multi-scale understanding of brain function: the global complexity of neural activity increases in conscious brain states so as to enhance responsiveness to stimuli. We suggest responsiveness may depend on the capacity of neural networks to transiently collapse the dimensionality of collective dynamics—in particular neural assemblies sensitive to stimulus features—into evoked low-dimensional trajectories supporting neural codes (Figure 1A).

## Macroscopic Signals Vary Robustly Between Brain States

Both spontaneous and evoked (Figures 1A,B) neural signals vary macroscopically across brain states, as demonstrated in electroencephalography (EEG), magnetoencephalography (MEG), and functional magnetic resonance imaging (fMRI). In unconscious states, neural activity is dominated by low-frequency, high-amplitude signals (Niedermeyer and Lopes da Silva, 2005). Accordingly, analyses of entropy (Sitt et al., 2014; Engemann et al., 2018), complexity (Tononi and Edelman, 1998), and dimensionality (El Boustani and Destexhe, 2010) during unconscious states indicate a relative simplicity of signals compared to conscious states. In unconscious states, synchronous activity slowly sweeps across the cortex (Massimini et al., 2004) along paths formed by cortical tracts (Capone et al., 2017). In both conscious resting and unconscious states, the default mode network (Raichle et al., 2001; Boly et al., 2008) establishes a pattern of synchronization between brain areas, producing correlations in ultra-slow (< 1 Hz) dynamics (Brookes et al., 2011). Sustained, slow oscillations were initially reported in the thalamocortical system (Steriade, 2003), but are also observed experimentally in isolated cortex, without thalamus (Sanchez-Vives and McCormick, 2000; Timofeev et al., 2000). Thalamocortical connections shape slow wave dynamics (Destexhe et al., 2007; Poulet et al., 2012; David et al., 2013; Crunelli et al., 2015; Zucca et al., 2019) although slow oscillations appear to be the default state of cortical networks (Sanchez-Vives and McCormick, 2000; Sanchez-Vives et al., 2017).

Patterns of neocortical regions activated in resting state networks have been successfully retrieved using eigenmodes of the structural connectivity matrix, i.e., the possible oscillatory patterns at frequencies allowed by white matter tract lengths (Atasoy et al., 2016). In active states, the executive control network replaces the default mode (Fox et al., 2005), and the co-activation of different cortical regions is more strongly controlled by correlations in external stimuli than by white matter structural connectivity (Gilson et al., 2018), with patterns of activity propagating recurrently between low-level, sensory areas and high-level, associative areas.

During conscious states, on the background of globally disorganized neural activity, transient patterns emerge (Duncan-Johnson and Donchin, 1982; Goodin and Aminoff, 1984; Sur and Sinha, 2009; Uhlhaas et al., 2009; Luck and Kappenman, 2011; Churchland et al., 2012; Sato et al., 2012; Singer, 2013; Chemla et al., 2019). Under an interpretation of brain states in analogy to states of matter, microscopic changes in the interactions between neurons could permit the emergence of larger-scale structures in brain activity.

## Microscopic Mechanisms; Biophysics of Brain States

**Figure 2:**
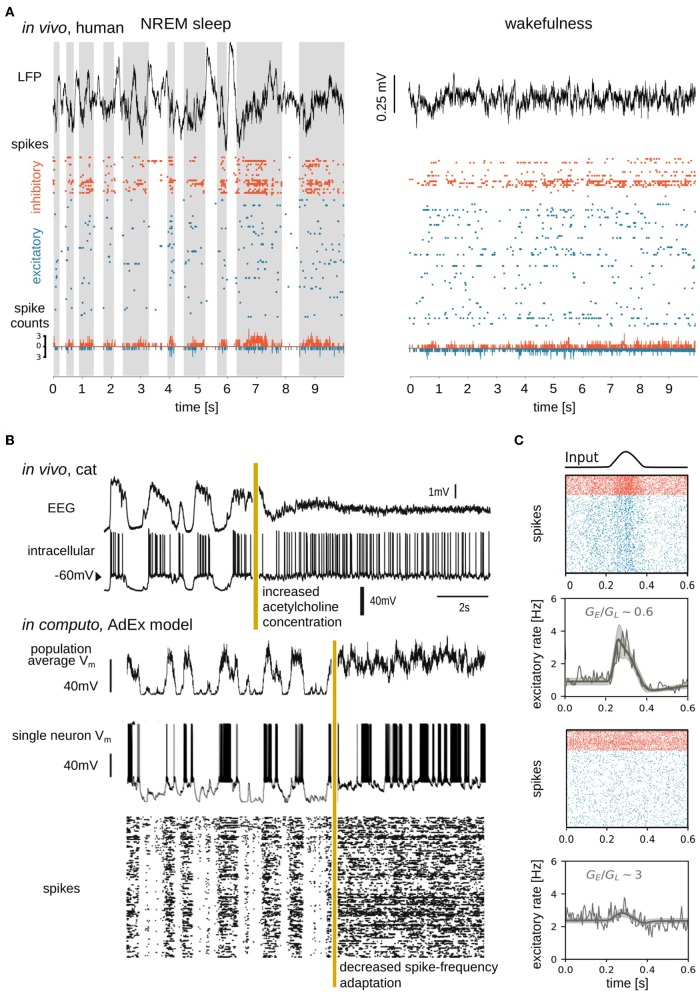
Simple, high-amplitude signals in unconscious brain states are associated with synchronous regular neuronal firing, whereas complex, low-amplitude signals in conscious brain states emerge from asynchronous irregular firing. **(A)** Data sample from Peyrache et al. (2012), Dehghani et al. (2016), Le Van Quyen et al. (2016), Teleńczuk et al. (2017), and Nghiem et al. (2018b), containing local field potential (LFP; top), spike times (action potentials; middle), and spike counts (bottom) recorded from a human subject during NREM sleep (left) and wakefulness (right). Spikes from inhibitory (orange) and excitatory (blue) neurons were separated and spike counts were calculated in bins of 5ms. Up states shaded in the left panel. **(B)** Transition between slow-wave (unconscious) and activated (conscious) state dynamics *in vivo* (top) and *in silico* (bottom). Experimentally the transition is generated by electrical stimulation of acetylcholine neurons in the pedunculopontine tegmentum (PPT) of anesthetized cat (Volgushev et al., 2011), triggering awake-like, desynchronized dynamics in cortex (Rudolph et al., 2005). A prominent consequence of enhancing cholinergic signaling in cortex is a reduction of spike-frequency adaptation (McCormick, 1992). *In silico*, a similarly desynchronizing effect can be generated by reducing the parameter responsible for spike-frequency adaptation. Simulated traces shown in the bottom were modified from Destexhe (2009), which used a network of adaptive exponential integrate-and-fire neurons. The average V_*m*_ of the network, the V_*m*_ of a randomly chosen neuron, and the raster plot of the network are shown. Reproduced with permission from Destexhe (2009). **(C)** State dependence of network responsiveness. The responsiveness of two spiking networks to a Gaussian pulse is shown. Raster plots display spike times of excitatory (blue) and inhibitory (orange) neurons connected by conductance-based synapses. Population activity (spike counts, thin line), as well as mean-field model (thick lines), and standard deviation (shaded area) of population firing rate generated by a mean-field model developed in di Volo et al. (2019). Responsiveness is found to vary between different network states, obtained by changing the ratio of the time-averaged global excitatory conductance (*G*_*E*_) (Destexhe et al., 2003) to membrane leakage conductance (*G*_*L*_).

Experiments have demonstrated that during unconscious brain states, the membrane potential (V_*m*_) of single cells slowly oscillates between hyperpolarized and depolarized potentials associated with alternating periods of silence (Down states, also termed “OFF periods”) and AI-like firing (Up states, also termed “ON periods”) (Steriade et al., 1993) (Figure 2A). During conscious brain states, neurons show sustained but sparse and irregular AI firing patterns (Vreeswijk and Sompolinsky, 1998; Destexhe et al., 1999; Brunel, 2000; Steriade, 2000; Renart et al., 2010; Dehghani et al., 2016; di Volo and Torcini, 2018). It was found that, during AI states, excitatory (E) and inhibitory (I) synaptic inputs are near-balanced (Dehghani et al., 2016), as predicted theoretically (Van Vreeswijk and Sompolinsky, 1996). In AI states, voltage fluctuations drive neurons over the threshold for firing action potentials, resulting in irregular spiking dynamics, also known as fluctuation-driven regimes (Kuhn et al., 2004; Destexhe, 2007; Destexhe and Rudolph-Lilith, 2012). To understand mechanisms at work during fluctuation-driven dynamics, computational models have further shown that three parameters are important to capture neuronal responses in this regime, the average membrane voltage *V*_*m*_, the amplitude of V_*m*_ fluctuations, and the conductance state of the membrane (Reig et al., 2015; Zerlaut et al., 2016).

Neuromodulators, including acetylcholine, play important biological roles in modulating the membrane properties of neurons (McCormick, 1992) and thus transitions between AI and slow oscillatory dynamics through the regulation of membrane currents (Hill and Tononi, 2005). Neuromodulators are present at higher concentrations during conscious states (McCormick, 1992; Jones, 2003) and, most generally, inhibit potassium (activity-dependent and leak *K*^+^) channels, which leads to depolarization of cells and suppression of *spike-frequency adaptation*. At low neuromodulatory concentrations, during unconscious states, *K*^+^ leak channels are constitutively open and activity-dependent *K*^+^ channels open when neurons spike, allowing *K*^+^ ions to exit cells thus hyperpolarizing the membrane. Accumulating self-inhibition in the form of spike-frequency adaptation during Up periods results in the transition to Down states. Conversely, spike-frequency adaptation wears off during Down states, allowing noise fluctuations (present ubiquitously; Destexhe and Rudolph-Lilith, 2012) to trigger transitions to Up states (Destexhe, 2009; Jercog et al., 2017; Nghiem T.-A. E. et al., 2018; di Volo et al., 2019) (Figure 2B). Computationally speaking, for high values of spike-frequency adaptation, bistability can be observed, with solutions at firing rate zero (Down state) and non-zero (Up state) values (Holcman and Tsodyks, 2006; di Volo et al., 2019). The more chaotic dynamics of AI states associated with consciousness allows for more reliable stimulus encoding (D'Andola et al., 2017), more reliable propagation (Zerlaut and Destexhe, 2017), and more sustained responses (Nghiem T.-A. E. et al., 2018) to stimuli over time. In contrast, during unconscious states, neuronal responses are more unreliable and vary greatly depending on the stimulus amplitude and whether cells receive inputs in Up or Down periods (Rosanova and Timofeev, 2005; Reig et al., 2015).

The Ising model for spin glasses (Jaynes, 1982) fitted to neural data (Schneidman et al., 2006) has revealed divergent types of emergent neuronal dynamics in conscious and unconscious states. While neuronal interactions are pairwise in wakefulness (Nghiem et al., 2017), coupling becomes population-wide in deep sleep (Tavoni et al., 2017; Nghiem et al., 2018b). In particular, inhibitory neurons organize synchronous activity across populations (Nghiem et al., 2018b; Zanoci et al., 2019), especially during deep sleep (Peyrache et al., 2012; Le Van Quyen et al., 2016; Olcese et al., 2016) where inhibitory neurons regulate rhythms of slow wave dynamics (Compte et al., 2008; Funk et al., 2017; Zucca et al., 2017, 2019).

To summarize, between unconscious and conscious brain states, microscopic data appear intuitively related to macroscopic data: synchronous microscopic Up and Down states resulting from constitutive and activity-dependent, hyperpolarizing currents due to reduced neuromodulation correspond to relatively simple, high-amplitude macroscopic dynamics observed in unconscious states. Active, disorganized, desynchronized, AI, low adaptation, high neuromodulation conditions correspond to low amplitude, complex, conscious brain signals. On backgrounds of differing spontaneous dynamics, generalizable patterns of activity (a.k.a. neural graphoelements) are observed. Cash et al. have elegantly shown that K-complexes (graphoelements characteristic of sleep stage 2) are complementarily observed both at microscopic and macroscopic scales (Cash et al., 2009). Other identifiable patterns also begin to emerge in empirical and theoretical data, including phase cones (Freeman and Barrie, 2000) and interacting traveling waves (Sato et al., 2012; Chemla et al., 2019). Since statistical physics has successfully described neuronal interactions for different brain states, we ask next whether mesoscale methods from statistical physics can help represent spontaneous and evoked dynamics of neuronal populations, thus formally linking knowledge between micro- and macroscopic scales.

## Mesoscale Bridges; Populations of Neurons

Brain dynamics at mesoscopic scales, describing thousands of neurons, are investigated empirically by electrophysiology and more recently, voltage-sensitive dyes (Arieli et al., 1996; Chemla and Chavane, 2010). At mesoscales, brain activity follows the trend of increasing complexity of spontaneous activity with consciousness (Figure 2A). Studying the effects of inputs at the mesoscale, studies have shown that perturbations during deep sleep states induce slow waves, but, during waking states, perturbations can result in chains of phase-locked activity (Pigorini et al., 2015) leading to causal global interactions (Rosanova et al., 2018).

*Mean-field* models offer a formalism for scaling up microscopic detail to collective macroscopic dynamics using few equations, offering a computational advantage for simulations. In describing states of matter, mean-field models simplify the probabilistic behavior of molecules to the relatively more predictable behavior of macroscopic states (Kadanoff, 2009). A rich literature has begun to develop mean-field models of neuronal populations, showing that global variables describing population activity can be usefully derived from the biophysics of neurons and their interactions (Ohira and Cowan, 1993; Ginzburg and Sompolinsky, 1994; El Boustani and Destexhe, 2009; Buice et al., 2010; Dahmen et al., 2016). Mean-field models have qualitatively reproduced temporal features of spontaneous dynamics including AI (El Boustani and Destexhe, 2009), Up and Down dynamics (Compte et al., 2003; Jercog et al., 2017; Tartaglia and Brunel, 2017; di Volo et al., 2019), and transitions between these states (di Volo et al., 2019; Tort-Colet et al., 2019). In addition, connecting mean-fields provides a tool for simulating the propagation of patterns through time and space, across mesoscale structures. For example, recent work deriving mean-field models of networks with conductance-based synapses has reproduced the suppressive interaction between traveling waves observed in visual cortex during conscious states, a biological phenomenon that could not be captured by current-based networks (Chemla et al., 2019).

Mean-field models have highlighted that, while complicated to apply mathematically in the framework of conductance-based models (di Volo et al., 2019), voltage-dependent interactions constitute a significant non-linearity in the membrane evolution equations. Voltage-dependent interactions appear to be important for explaining non-trivial responses of biological neurons, through the mean and fluctuations of the cells' membrane voltage (Reig et al., 2015). In fact, while these results do not imply that differences in responsiveness are due only to conductances, they show that voltage dependent synapses play a role in the nonlinear state-dependent response of a neural network. As shown in Figure 2C, various levels of membrane conductance, regulated by voltage-dependent synapses, are shown to differently shape population responses.

Finally, renormalization group theory, a method of coarse-graining microscopic detail to obtain macroscopic laws helping to understand how order can emerge from apparent disorder (Wilson, 1979; Cardy, 1996; Goldenfeld, 2018) has recently begun to be applied to neural assemblies (Meshulam et al., 2019), laying further foundation for the formal connection of our understanding of brain function across scales.

## Discussion

In this paper, we briefly reviewed work on the measurement and modeling of brain states at different scales, from single neurons to cell assemblies and global brain activity, considering both spontaneous and evoked dynamics. In particular, we highlighted that increased complexity in the dynamics of conscious brain states relates to changes in single-neuron biophysics, tuned by neuromodulation. In unconscious states, reduced neuromodulation promotes activity-dependent self-inhibition of excitatory neurons as they spike, leading to alternating, synchronous transients of silence and firing, that produce high-amplitude, low-complexity, synchronous signals, on resonant frequencies of the structural connectome. During conscious states, neuronal discharges are asynchronous, irregular and fluctuation-driven, resulting from sustained membrane depolarization in cortical neurons, promoting effective neural communication.

Beyond conscious and unconscious categories proposed here for the sake of brevity, important differences exist within categories of unconscious and conscious states (Brown et al., 2010; El Boustani and Destexhe, 2010; Nghiem et al., 2018a). Unlike healthy wakefulness and sleep, epileptic networks display both excessively high conductance and strongly synchronized, regular dynamics (El Boustani and Destexhe, 2010). Further, brain signals in coma are both low-amplitude and low-complexity, in contrast to high-amplitude signals observed in other unconscious states, but also to complex signals observed in conscious states (El Boustani and Destexhe, 2010). Such anomalous deviations from the overall trend of coordinated changes in complexity and amplitude may illuminate mechanisms underlying disease-causing deviations from healthy brain states (Mackey and Glass, 1977).

To characterize brain states, it has been useful to consider not only spontaneous dynamics but also patterns evoked by perturbations. It was found that macroscopic responsiveness highly depends on brain state and different patterns of responses are evoked in conscious versus unconscious states (Massimini et al., 2005). Such state-dependent responsiveness can also be seen at the level of local networks *in vivo* and *in silico*, for example in the different reliability of responses to perturbations given during Up and Down periods of slow waves (Reig et al., 2015; Zerlaut and Destexhe, 2017). In simulations, different responsiveness could be accounted for by three parameters: membrane voltage, voltage fluctuation amplitude, and membrane conductance (Reig et al., 2015). These parameters could be well described by mean-field models (di Volo et al., 2019), able to capture fundamental properties of spontaneous dynamics and also state-dependent responses at mesoscales. As such, the data-driven coupling of such mean-field models may serve as natural candidates for modeling the emergence of mesoscopic and macroscopic-scale patterns.

Transient collapses of dimensionality found in encoding networks were also discussed as substrates potentially supporting neural codes. Such collapses in complexity have been observed in active ensembles at scales spanning microscopic (Churchland et al., 2010; Fairhall, 2019) to macroscopic (Quiroga et al., 2001; Zang et al., 2004) activity. This echoes recent work studying recordings of neural populations which highlighted that neural representations of stimuli may lie on low-dimensional manifolds (Churchland et al., 2012; Sadtler et al., 2014; Gallego et al., 2017; Zhao and Park, 2017; Golub et al., 2018; Chaudhuri et al., 2019; Recanatesi et al., 2019; Stringer et al., 2019). Indeed neurons do not fire independently, which would yield dynamics of dimensionality as high as the number of neurons, but instead follow constrained trajectories of activity that can be captured by descriptions of much lower dimensionality that depend on spontaneous and evoked dynamics. For example, a neural population firing in synchrony could be fully described by a periodic orbit trajectory constrained to a low-dimensional space (Churchland et al., 2012). Since spontaneous global network activity increases in dimensionality during conscious states, we ask whether the transient collapse of complexity in specific networks, translating the emergence of simpler dynamical structures from disorder, may be associated to neural codes.

As an analogy, windmills facing all in one direction display low complexity, but can only be synchronously active or inactive. Windmills facing in random directions, in contrast, are a higher complexity configuration able to represent wind from any direction through the activity of a subset. The activity of an ensemble of windmills tuned to a particular direction of wind could represent a collapse of complexity and the generation of information by that subset (in this case, about the direction of wind). Similarly, enhanced dimensionality associated with conscious states could subserve neural information through the collapse of complexity in neural assemblies tuned to encode particular representations.

## Author Contributions

All authors listed have made a substantial, direct and intellectual contribution to the work, and approved it for publication.

### Conflict of Interest

The authors declare that the research was conducted in the absence of any commercial or financial relationships that could be construed as a potential conflict of interest.
